# Association between Nocturnal Sleep Duration and Insomnia symptoms with depressive symptoms among 44,900 Chinese Han adults aged 30–79 in Southwest China

**DOI:** 10.1186/s12888-023-04601-6

**Published:** 2023-02-27

**Authors:** Yang Gao, Wenge Tang, Deqiang Mao, Liling Chen, Xianbin Ding

**Affiliations:** Chongqing Center for Disease Control and Prevention, 400042 Chongqing, China

**Keywords:** Depressive symptoms, Nocturnal sleep duration, Insomnia, Chinese Han adults

## Abstract

**Background:**

Although there are several cross-sectional and prospective studies on the relationship between sleep duration /insomnia symptoms and depression symptoms, the results of these studies are still not conclusive, and few studies have further analyzed the association between sleep duration and depressive symptoms in adults by gender and age. Thus, this study aimed to investigate the relationship between nocturnal sleep duration and insomnia symptoms with depression symptoms, and further examine whether the association was impacted by age and gender in a large-scale Han Chinese population in southwest China.

**Methods:**

A cross-sectional study was performed that included 44,900 participants from 18 districts in southwest China from September 2018 to January 2019. The study comprised 42,242 individuals in the final analysis. Depressive symptoms were investigated using the PHQ-2 questionnaires. Multivariate logistic regression analysis was performed to investigate the relationship between nocturnal sleep duration and insomnia symptoms with depression symptoms as well as the influence of age and gender.

**Results:**

After adjusting for multiple variables, those with nocturnal sleep duration < 7 h had a higher odds ratio for depression (OR:1.47, 95%CI 1.31–1.65) compared to participants whose nocturnal sleep duration was in the range of 7-8 h. Notably, there is a higher association in those aged below 45 years (OR:1.91, 95%CI 1.52–2.41) and in female participants (OR:1.57, 95%CI 1.35–1.82). However, nocturnal sleep duration longer than 9 h was not associated with depression symptoms in either the whole population analysis or the subgroup analysis. Insomniacs had a higher odds ratio for depression(OR:1.87, 95%CI 1.84–2.36, respectively) compared to non-insomniacs. There is a higher association in those aged 45–60 years (OR:2.23, 95%CI 1.82–2.73) and in female participants (OR:2.17, 95%CI 1.84–2.56). Further subgroup analysis by age and gender at the same time showed the association between sleep deprivation and depression was highest among women aged below 45 years, while the association between insomnia and depression was the highest among men aged 45–59 years.

**Conclusion:**

Short nocturnal sleep duration and insomnia symptoms were positively associated with the risk of depressive symptoms among Chinese Han adults aged 30–79 in Southwest China, especially the middle-aged population and females should be more concerned.

## Introduction

Depression is a common mental disorder and a major cause of disability. The 2017 Global Burden of Disease Report states that more than 264 million people suffer from depression globally [1]. Depression can seriously impair social function, increase psychosocial disability, and reduce the quality of life in individuals of all ages. The prevention and treatment of depression have become an important public health issue worldwide. Sleep, as a very important health-related factor, has recently attracted increasing attention from scholars. Studies showed that sleep duration has a significant impact on total mortality, cardiovascular disease, metabolic syndrome, and other common chronic diseases [2–4]. Insomnia is one of the most common sleep disorders in the world, and the main symptoms include having difficulty falling asleep at night, waking up during the night, or waking up too early [5, 6]. Numerous studies have found that insomnia is linked with many detrimental health outcomes, such as rheumatoid arthritis, myocardial infarction, coronary heart disease, and stroke [7, 8]. Insomnia is also a significant risk factor for several mental disorders, especially depression and anxiety [7]. Although the association between sleep duration and insomnia with depressive symptoms has been examined, the results of these studies are still not conclusive.

Various studies have demonstrated that either shorter or longer sleep duration was associated with an increased risk of depressive symptoms [9–11]. However, some studies only concluded that insufficient sleep duration was associated with depression symptoms, and did not find an association between excessive sleep duration and depression [12, 13]. Therefore, the relationship between sleep duration and depressive symptoms needs further research to confirm.

Although there were several cross-sectional and prospective studies on the relationship between insomnia symptoms and depression symptoms [7, 14–16], few studies have further analyzed the association between insomnia symptoms and depressive symptoms in adults by gender and age. Epidemiological studies have consistently shown a higher prevalence of insomnia symptoms and short sleep duration among women compared to men [17], while the rate of short sleep duration increases with age [18]. Thus, we hypothesize that the relationship between sleep and depressive symptoms will also present vary across age and gender.

Based on the insights above, the present study aimed to investigate the relationship between nocturnal sleep duration and insomnia symptoms with depression symptoms, and further examine whether the association was impacted by age and gender in a large-scale Han Chinese population in southwest China.

## Materials and methods

### Study participants

**Fig. 1:**
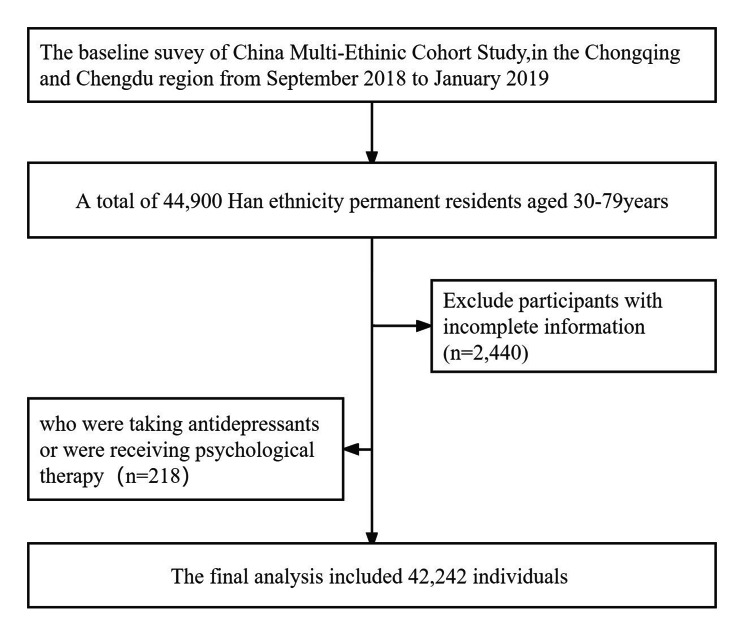
Data cleaning flowchart

This cross-sectional study was based on the China Multi-Ethnic Cohort(CMEC) Study. Detailed information about the CMEC study design, survey methods and population have been described in a previous report [19]. The data utilized in the current study were obtained from the Sichuan Basin region, two (Chongqing and Sichuan provinces) of the 5 regions included in the CMEC study. In brief, 44,900 Chinese Han participants aged 30–79 years were recruited and participated in the baseline survey from September 2018 to February 2019. This population-based survey was carried out in 18 districts/counties by a multistage, stratified cluster sampling from Chongqing (the district and county are of the same administrative level in Chongqing), and Sichuan provinces, including Yuzhong District, Jiulongpo District, Nanan District, Banan District, Changshou District, Jiangjin District, Hechuan District, Qijiang District, Dazu District, Tongnan District, Rongchang District, Wulong District, Fengdu County, Chenghua District, Qingbaijiang District, Wuhou District, Pidu District and Jianyang city. Electronic questionnaires and health exams were mainly applied to collect participant data, including demographic and socioeconomic details, health behaviors, disease history, mental health status, and insomnia symptoms. Participants were excluded if they had incomplete information (n = 2440), were taking antidepressants, or receiving psychological therapy(n = 218). Therefore, the final analysis comprised 42,242 individuals to evaluate the association between sleep and depression symptoms. (Fig. 1). All the participants signed an informed consent form before data collection. This survey was approved by the ethics committee of Sichuan University (No. 78K2016038).

### Data Collection and Laboratory Measurement

Questionnaire information was collected through face-to-face interviews using a tablet with automatic recording capabilities (CMES app). The entire interview is conducted by trained interviewers who are either medical staff at a local medical facility or medical students at a local university. The content of the questionnaire includes socioeconomic status (gender, age, marital status, education, occupation, annual family income), lifestyle habits ( alcohol drinking, smoking, physical activity), self-rated health status, pain rate, social capital, stressful life events, history of hypertension, history of diabetes, BMI(body mass index), night sleep duration, income, and psychological conditions.

### Assessment of nocturnal sleep duration

Information on nocturnal sleep duration was collected in face-to-face interviews by trained interviewers. Nocturnal sleep duration was derived from the answer to the question, “How many hours did you usually sleep at night in the past month?” Self-reported nocturnal sleep duration was categorized into < 7, 7–8, and ≥ 9 h groups. Based on the recommendations of the National Sleep Foundation [20], the sleep duration of 7 to 8 h was used as the reference group.

### Insomnia symptoms

CMEC includes the Sleep Quality Assessment Scale. Three symptoms of insomnia were measured in the Sleep Quality Index last month:1) Difficult to fall asleep at night (≥ 30 min) for 3 or more days a week; 2)Waking up prematurely and having difficulty returning asleep 3 or more days per week; and 3) having to take medicine to help sleep more than one day a week. If participants answered “yes” to any of these three questions, we classified it as a symptom of insomnia. The cut-offs for insomnia were based on the criteria in the Diagnostic and Statistical Manual of Mental Disorders(DSM-V ) [21].

### Assessment of depressive symptoms

Depressive symptoms were assessed using the PHQ-2 questionnaire, which is a two-screen questionnaire based on the Diagnostic and Statistical Manual of Mental Disorders Fourth Edition (DSM-IV) that assesses depressive symptoms over the past two weeks with the following two questions: (1) little interest or pleasure in doing.

things, and (2) feeling down, depressed, or hopeless (PHQ-2). For each item, the response options are “Not at all,” “Several days,” “More than half of the days, ” and “Nearly every day, ” corresponding to a score of 0, 1, 2, and 3. The total score of the PHQ-2 ranges from 0 to 6. A cutoff of 3 was adopted to identify depressive symptoms. This ultrashort screening tool considerably enhances the efficiency of screening and monitoring depressive symptoms during intense primary care practices and large population-based epidemic surveys [22–24].

### Confounding factors

Confounding factors include demographic and socioeconomic information, lifestyle habits, health-related variables, social capital factors, and stressful life events.

Demographics encompassed gender, age, and registration (rural or urban). Socioeconomic status was determined by marital status (married or living with a partner or others ), educational level ( primary school or below, junior high school, high school or junior college and above), occupation(employed, retired, or unemployed), and yearly household income (< 12,000 yuan, 12,000–19,999 yuan, 20,000–59,999 yuan, 60,000–99,999 yuan or ≥ 100,000 yuan). Lifestyle habits contained alcohol drinking status(never or hardly, occasionally, regularly ), smoking status (never, smoking or quit smoking ), and physical activity level was estimated based on metabolic equivalent (MET) with a dichotomized variable using 18.62 h/day as the cutoff point according to the median(≤ 18.62 or > 18.62).Health-related variables included body mass index (BMI) categories (< 24, 24–27.9 or ≥ 28 kg/m^2^), self-assessed health status (good moderate, bad), self-reported physical pain or discomfort rating (ranging from 1 to 5; 1 = no pain or discomfort and 5 = severe pain or discomfort), history of hypertension(no or yes), history of diabetes(no or yes).

Social capital was measured using a validated Chinese version of Health-related Social Capital Measurement [25, 26]. The Family Social Capital Scale is divided into two categories: (1) “Experiential Family Support” and (2) “Consistently Obtaining Financial Support from Family”. The Community and Social Social Capital Scale uses three items: (1) “Frequency of participation in community organization activities in the previous year” and (2) “Activity supported by community organizations in the previous year”. (3) “You’ve been treated fairly by society”. The answer categories ranged from a scale of 1 (strongly disagree) to 5(strongly agree), with higher total scores, indicating stronger social capital. Stressful life events were assessed by following 10 stressful events during the past two years:1)divorce/separation; 2) loss of job/retirement; 3) business failure or bankruptcy; (4) being violently attacked/raped; (5) serious family internal contradictions and conflicts; (6) a serious injury or car accident; (7) serious illness or death of a spouse; (8) serious illness or death of other close family members; (9) serious natural disasters (e.g., drought or flood); (10) loss of the source of income/living in debt [27]. If a participant answered ‘yes’ to any of those 10 stressful events, we classified him/her as having stressful life events.

### Statistical analysis

Statistical analyses were performed using the Statistical Program for Social Sciences (SPSS)version 25.0 (SPSS Inc., Chicago, IL, USA). All continuous data are described as mean ± standard deviation, and categorical data are summarized as percentages. One-way ANOVA was used to compare differences in continuous data, and chi-square tests were used to compare differences in categorical data. Logistic regression was applied to test the association between sleep and depression symptoms after adjustment for potential confounders, and the odds ratio (OR) and 95% confidence interval (CI) for the outcome variable were calculated. The final model included eighteen covariates (gender, age, registration, marital, education level, occupation, annual family income, alcohol drinking status, smoking status, physical activity,self-rated health status, pain rate, family social capital, community/society social capital, stressful life events, history of hypertension, history of diabetes, and BMI). We conducted additional stratified analyses to examine the modification effect of age(**< 45, 45–59, and** ≥ **60**), and gender(male and female). All statistical tests were two-sided and a P value < 0.05 was considered statistically significant.

## Results

### General characteristics of the participants

Table 1 outlines the participants’ basic characteristics. The mean age of those with depression symptoms was older than that of participants without depressive symptoms. 54.3% of the total population were female; who had a higher prevalence of depression symptoms than their male counterparts. The education level of Residents with depression symptoms were generally lower than their peers without depression symptoms (49.4% vs. 68.6% for junior high school and above). Residents with depression symptoms had lower incomes than those without depression symptoms (63.7% vs. 78.1% for an annual family income ≥ 20,000 yuan). Smoking was reported more frequently by participants with depression symptoms, but Alcohol drinking was reported less frequently by those with depression symptoms (29.7% vs. 27.7% for smoking or,14.0% vs. 16.3% for drinking regularly). The depressed tended to have less Physical activity non-depressed (46.8% vs.50.1% for physical activity higher than 18.62MET hour/day).

The depressed reported higher pain ratings and worse self-assessed health than the non-depressed (20.00% vs. 3.67% for a pain rating ≥ 3; 31.9% vs. 5.2% for bad self-assessed health). The mean scores for family social capital and community and.

society social capital was reported higher by participants with depression symptoms (5.78vs.4.64 for family Sc, 9.07vs.8.08 for community and Society SC). Participants with depression symptoms reported a higher rate of stressful life events than participants with non-depressive symptoms (52.3% vs.28.2%for having stressful life events at least once). A higher share of depressed than non-depressed had a history of hypertension and diabetes (38.6%vs. 33.0% for hypertension; 16.0% vs. 12.2% for diabetes). The depressed had a higher prevalence of obesity (15.7% vs. 13.8% for BMI ≥ 28 kg/m2). The depressed group had higher levels of insomnia symptoms and shorter nocturnal sleep duration (75.8% vs. 44.3% for insomnia,51.7% vs. 29.2% for nocturnal sleep duration < 7 h).

**Table 1:** Sociodemographic characteristics and selected characteristics by depressive symptoms

Variable	Depressive symptoms			*P* value
	Total (n = 42,242)	No(N = 40,525 )	Yes(N = 1717 )	
Gender, n(%)				<0.001
Male	19,291(45.7%)	18,606 (45.9%)	685 (39.9%)	
Female	22,951(54.3%)	21,919 (54.1%)	1032 (60.1%)	
Age(year)(SD)	51.3 ± 12.0	51.3 ± 12.0	54.6 ± 12.6	<0.001
Registration, n (%)				<0.001
Rural	17,098 (40.5%)	16,210 (40.0%)	888 (51.7%)	
Urban	25,144 (59.5%)	24,315 (60.0%)	829 (48.3%)	
Marital, n (%)				<0.001
Married or cohabitation	37,581 (89.0%)	36,158 (89.2%)	1423 (82.9%)	
Others	4661 (11.0%)	4367 (10.8%)	294 (17.1%)	
Education level, n (%)				<0.001
Primary or below	13,575 (32.1%)	12,707 (31.4%)	868 (50.6%)	
Junior high school	13,598 (32.2%)	13,137 (32.4%)	461 (26.8%)	
High school	7617 (18.0%)	7406 (18.3%)	211 (12.3%)	
Junior college and above	7452 (17.6%)	7275 (18.0%)	177 (10.3%)	
Occupation, n (%)				<0.001
Employed	26,911 (63.7%)	25,913 (63.9%)	998 (58.1%)	
Retirement	6777 (16.0%)	6564 (16.2%)	213 (12.4%)	
Unemployed	8554 (20.2%)	8048 (19.9%)	506 (29.5%)	
Annual family income, yuan (%)				<0.001
< 12,000	4322 (10.2%)	3943 (9.7%)	379 (22.1%)	
12,000–19,999	5175 (12.3%)	4931 (12.2%)	244 (14.2%)	
20,000–59,999	15,056 (35.6%)	14,421 (35.6%)	635 (37.0%)	
60,000–99,999	8877 (21.0%)	8627 (21.3%)	250 (14.6%)	
≥ 100,000	8812 (20.9%)	8603 (21.2%)	209 (12.2%)	
Alcohol drinking status, n (%)				<0.001
Never or hardly	19,761 (46.8%)	18,834 (46.5%)	927 (54.0%)	
Occasionally	15,627 (37.0%)	15,077 (37.2%)	550 (32.0%)	
Regularly	6854 (16.2%)	6614 (16.3%)	240 (14.0%)	
Smoking status, n(%)				0.078
Never	29,739 (70.4%)	28,497 (70.3%)	1242 (72.3%)	
Smoking or quit smoking	12,503 (29.6%)	12,028 (29.7%)	475 (27.7%)	
Physical activity (MET hour/day), n (%)				<0.01
≤ 18.62	21,120(50%)	20,206(49.9%)	914(53.2%)	
> 18.62	21,120(50%)	20,319(50.1%)	803(46.8%)	
Self-rated health status, n (%)				<0.001
Good	20,177 (47.8%)	19,890 (49.1%)	287 (16.7%)	
Moderate	19,418 (46.0%)	18,536 (45.7%)	882 (51.4%)	
Bad	2647 (6.27%)	2099 (5.2%)	548 (31.9%)	
Pain rate: n (%)				<0.001
1	31,369 (74.3%)	30,642 (75.6%)	727 (42.3%)	
2	9041 (21.4%)	8395 (20.7%)	646 (37.6%)	
3	1285 (3.04%)	1104 (2.72%)	181 (10.5%)	
4	514 (1.22%)	369 (0.91%)	145 (8.44%)	
5	33 (0.08%)	15 (0.04%)	18 (1.05%)	
Family social capital: mean (SD)	4.68 (2.49)	4.64 (2.47)	5.78 (2.64)	<0.001
Community/Society social capital: mean (SD)	8.12 (2.32)	8.08 (2.30)	9.07 (2.50)	<0.001
Stressful life events, n (%)				<0.001
No	29,927 (70.8%)	29,108 (71.8%)	819 (47.7%)	
Yes	12,315 (29.2%)	11,417 (28.2%)	898 (52.3%)	
Hypertension, n (%)				<0.001
No	28,208 (66.8%)	27,153 (67.0%)	1055 (61.4%)	
yes	14,034 (33.2%)	13,372 (33.0%)	662 (38.6%)	
Diabetes, n (%)				<0.001
No	37,017 (87.6%)	35,575 (87.8%)	1442 (84.0%)	
yes	5225 (12.4%)	4950 (12.2%)	275 (16.0%)	
BMI (kg/m2): mean (SD)				<0.05
< 23.9	19,568(46.4%)	18,764(46.4%)	804(46.9%)	
24-27.9	16,754(39.7%)	16,113(39.8%)	641(37.4%)	
≥ 28	5854(13.9%)	5585(13.8%)	269(15.7%)	
Insomnia, n(%)				<0.001
No	23,006 (54.5%)	22,591 (55.7%)	415 (24.2%)	
yes	19,236 (45.5%)	17,934 (44.3%)	1302 (75.8%)	
Night sleep duration, h(%)				<0.001
< 7 h	12,719 (30.1%)	11,832 (29.2%)	887 (51.7%)	
7–9	25,328 (60.0%)	24,635 (60.8%)	693 (40.4%)	
≥ 9	4195 (9.93%)	4058 (10.0%)	137 (7.98%)	

Continuous data were described as mean ± standard deviations, and statistical significance was assessed by the one-way analysis of variance.

Categorical data were summarized as percentages (%), and statistical significance was assessed by chi-square test.

### Prevalence of depressive symptoms by age and sex

**Fig. 2:**
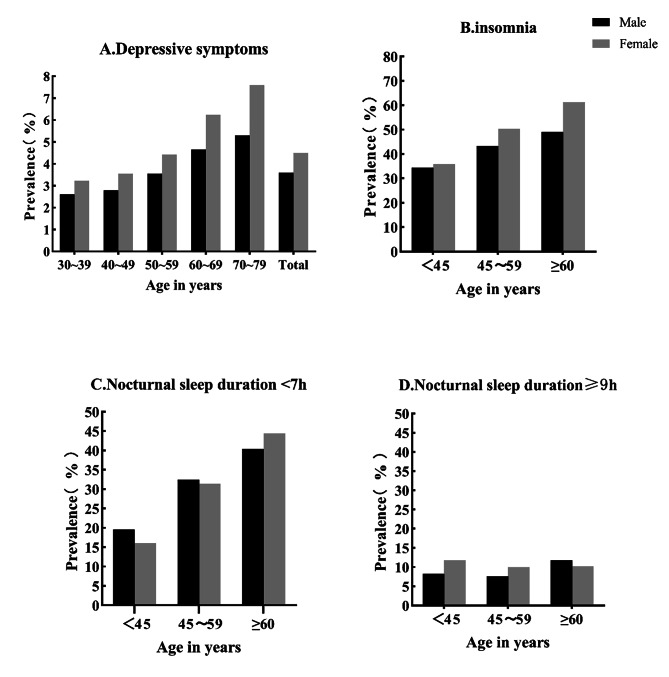
(A)Prevalence of depressive symptoms by age and sex (B ~ D) Distribution of nocturnal sleep duration and insomnia by age and sex

Figure 2 displays the prevalence of depressive symptoms, insomnia symptoms, nocturnal sleep time < 7 h and nocturnal sleep time ≥ 9 h. Overall, 4.1% of participants reported having depressive symptoms, which was 3.6% among men and 4.5% among women. The prevalence of depression increased with age in both men and women, and was higher in women than in men in all age groups. 45.5% of participants had insomnia, with a higher percentage of females (48.3%) than males (42.3%) increasing with age. 30.1% of participants slept < 7 h at night, with a higher percentage of men than women, increasing with age. 9.9% of participants slept ≥ 9 h at night, higher in males than females and highest in the age group ≥ 60 years.

### Association between nocturnal sleep duration and depression

**Fig. 3:**
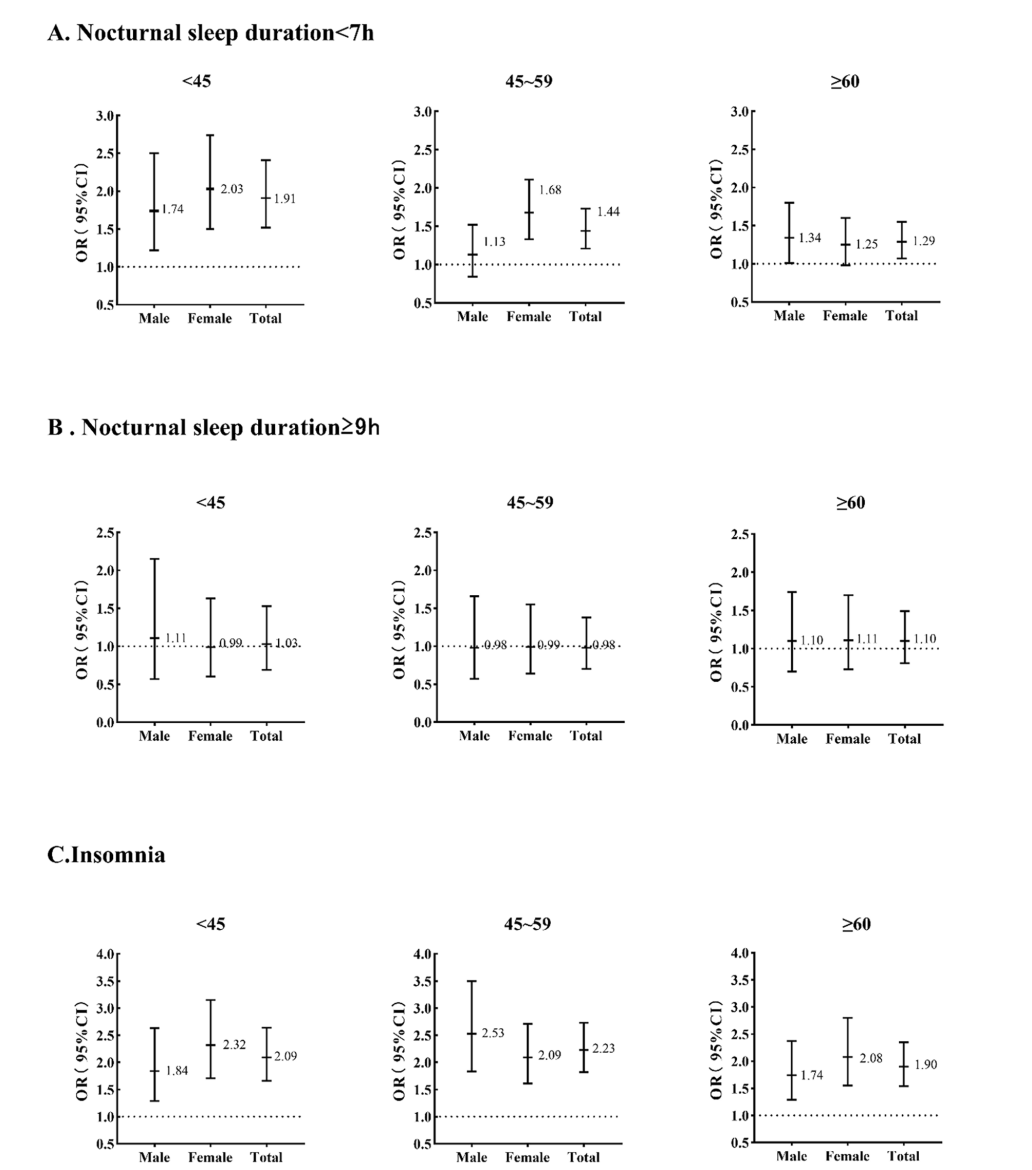
Odds ratio(OR) and 95% CI for depressive symptoms to binary categories (Males vs. Females) in three different age groups. (A)Results of nocturnal sleep duration < 7 h. (B)Results of nocturnal sleep duration ≥ 9 h. (C)Results of insomnia

Table 2; Fig. 3 display the association between nocturnal sleep duration and depression among all participants. In models 1 and 2, those with NSD < 7 h had a higher odds ratio for depression symptoms(OR:2.34, 95%CI 2.11–2.60; OR:1.47, 95%CI 1.31–1.65, respectively) compared to participants with 7 h ≤ NSD < 9 h.

Further subgroup analysis was performed separately for the age and gender of the recruits. As shown in the Table 2, the associations between short sleep duration (nocturnal sleep < 7 h )and depression symptoms showed significantly positive statistically in all three age groups and both males and females. Notably, there is a higher association in those aged less than 45 years(OR:1.91, 95%CI 1.52–2.41) and in female participants (OR:1.57, 95%CI 1.35–1.82). Moreover, we conducted stratification analysis by age and gender at the same time. Figure 3 shows that the association between short sleep duration and depression was highest among women aged less than 45 years, while the association was not statistically significant among men aged 45–59 years.

However, nocturnal sleep duration longer than 9 h was not associated with depression symptoms in either the whole population analysis or the subgroup analysis.

**Table 2:** Association between Nocturnal Sleep Duration and Insomnia with Depressive Symptoms

Variables	sleep duration								Insomnia			
	7~<9 h		< 7 h			≥ 9 h			No		Yes	
	OR(95%CI)		OR(95%CI)	***P*** **value**		OR(95%CI)	***P*** **value**		OR(95%CI)		OR(95%CI)	***P*** **value**
**Total**												
Crude model	1.00(reference)		2.67(2.41, 2.95)	< 0.01		1.20(0.99, 1.45)	0.06		1.00(reference)		3.95(3.53, 4.42)	< 0.01
Model 1	1.00(reference)		2.34(2.11, 2.60)	< 0.01		1.01(0.84, 1.22)	0.08		1.00(reference)		3.45(3.08, 3.87)	< 0.01
Model 2	1.00(reference)		1.47(1.31, 1.65)	< 0.01		1.06(0.88, 1.29)	0.06		1.00(reference)		2.08(1.84, 2.36)	< 0.01
**Age**												
**<45**												
Crude model	1.00(reference)		3.38(2.74, 4.17)	< 0.01		1.04(0.71, 1.53)	0.02		1.00(reference)		4.09(3.31, 5.05)	< 0.01
Model 1	1.00(reference)		3.17(2.56, 3.92)	< 0.01		0.94(0.64, 1.38)	0.74		1.00(reference)		3.79(3.06, 4.70)	< 0.01
Model 2	1.00(reference)		1.91(1.52, 2.41)	< 0.01		1.03(0.69, 1.53)	0.89		1.00(reference)		2.09(1.66, 2.64)	< 0.01
**45–59**												
Crude model	1.00(reference)		2.51(2.13, 2.96)	< 0.01		1.11(0.8, 1.53)	0.54		1.00(reference)		4.00(3.32, 4.82)	< 0.01
Model 1	1.00(reference)		2.39(2.02, 2.82)	< 0.01		0.96(0.69, 1.33)	0.79		1.00(reference)		3.59(2.97, 4.33)	< 0.01
Model 2	1.00(reference)		1.44(1.21, 1.73)	< 0.01		0.98(0.7, 1.38)	0.92		1.00(reference)		2.23(1.82, 2.73)	< 0.01
≥**60**												
Crude model	1.00(reference)		1.97(1.66, 2.33)	< 0.01		1.20(0.90, 1.60)	0.23		1.00(reference)		3.26(2.69, 3.94)	< 0.01
Model 1	1.00(reference)		1.90(1.60, 2.26)	< 0.01		1.04(0.78, 1.40)	0.78		1.00(reference)		3.01(2.48, 3.65)	< 0.01
Model 2	1.00(reference)		1.29(1.07, 1.55)	< 0.05		1.10(0.81, 1.49)	0.56		1.00(reference)		1.90(1.54, 2.35)	< 0.01
**Gender**												
**Male**												
Crude model	1.00(reference)		2.42(2.06, 2.84)	< 0.01		1.39(1.05, 1.85)	0.02		1.00(reference)		3.70(3.12, 4.38)	< 0.01
Model 1	1.00(reference)		2.12(1.80, 2.50)	< 0.01		1.11(0.83, 1.49)	0.46		1.00(reference)		3.28(2.76, 3.90)	< 0.01
Model 2	1.00(reference)		1.33(1.12, 1.60)	< 0.01		1.08(0.8, 1.47)	0.61		1.00(reference)		1.33(1.12, 1.60)	< 0.01
**Female**												
Crude model	1.00(reference)		2.86(2.51, 3.27)	< 0.01		1.07(0.84, 1.37)	0.57		1.00(reference)		4.09(3.52, 4.75)	< 0.01
Model 1	1.00(reference)		2.50(2.18, 2.86)	< 0.01		0.96(0.75, 1.23)	0.73		1.00(reference)		3.60(3.09, 4.20)	< 0.01
Model 2	1.00(reference)		1.57(1.35, 1.82)	< 0.01		1.06(0.82, 1.37)	0.65		1.00(reference)		2.17(1.84, 2.56)	< 0.01

Model 1: unadjusted model

Model 2: adjusted for gender, age, registration, marital, education level, occupation, annual family income, alcohol drinking status, smoking status, and physical activity

Model 3: adjusted for gender, age, registration, marital, education level, occupation, annual family income, alcohol drinking status, smoking status, physical activity, self-rated health status, pain rate, family social capital, community/society social capital, stressful life events, history of hypertension, history of diabetes, and BMI

### Association between insomnia and depression

Table 2; Fig. 3 display the association between insomnia and depression among all study participants. In models 1 and 2, the people with insomnia symptoms had a higher odds ratio for depression symptoms(OR:3.45, 95%CI 3.08–3.87; OR:1.87, 95%CI 1.84–2.36, respectively) compared to the one with no insomnia symptoms. Further subgroup analysis was performed separately for the age of recruitment and gender. As shown in the Table 2, the associations between insomnia symptoms and depression symptoms were significantly positive statistically in all three age groups and both males and females. Notably, there is a stronger association in those aged 45–60 years(OR:2.23, 95%CI 1.82–2.73) and in female participants (OR:2.17, 95%CI 1.84–2.56). Moreover, we conducted stratification analysis by age and gender at the same time. Figure 3 shows that the association between insomnia symptoms and depression symptoms in men aged 45–59 years was the highest.

## Discussion

In this large population-based study of adults aged 30–79 years in the Sichuan Basin, 4.1% of participants reported having depressive symptoms. By Comparing the prevalence of 6.4–38.7% of depressive symptoms with a few previous studies [9, 18, 28], the total prevalence in our study was low. The low prevalence could be underestimated because of the lack of mental health literacy and stigma-induced conscious non-disclosure reporting in the low-middle developing areas. In our study, we found that nocturnal sleep duration and insomnia were associated with depressive symptoms among adults aged 30–79 years. After adjusting for demographic and socioeconomic information, health behavior, health-related variables, and social capital factors, those who reported “nocturnal sleep < 7 hours” and “had insomnia symptoms ” had 47% and 87% increased risk of depression symptoms compared with “7 hours ≤ nocturnal sleep < 9hours” and “non-insomniacs”. However, Nocturnal sleep of 9 hours or longer was not associated with depression symptoms in either the whole population analysis or the subgroup analysis.

The study showed that nocturnal sleep duration and insomnia were associated with depressive symptoms among adults aged 30–79 years. After adjusting for demographic and socioeconomic information, health behavior, health-related variables, and social capital factors, those who reported“nocturnal sleep < 7 hours” and “had insomnia symptoms” had 47% and 87% higher risks of depression symptoms compared with“7 h ≤ nocturnal sleep < 9 h” and “non-insomniacs”. Furthermore, the effect of this association was higher in women. However, Nocturnal sleep of 9 h or longer was not associated with depression symptoms in either the whole population analysis or the subgroup analysis.

Our findings on the association between nocturnal sleep duration and depressive symptoms are consistent with some, but not all previous studies. The shorter or reduced duration of sleep has been shown to be a predictive factor for depression in some longitudinal or prospective studies [29]. A recent meta-analysis [30] including seven prospective studies indicated an association between short sleep duration and the risk of depression, with a risk ratio of 1.31 compared with normal sleep duration. Most studies on long sleep duration found no significant association between long sleep duration and depression [31–33], which were similar to the results of our study, while a meta-analysis suggested that longer sleep duration predicted an increased risk of depression [5]. Furthermore, some cross-sectional studies found that shorter (≤ 6 h ) or longer (≥ 9 h ) sleep duration was associated with an increased risk of depressive symptoms [9, 18, 34] and these also didn’t find significant associations between long sleep duration and depression symptoms [28, 35]. Potential reasons for the conflicting results could be that: (1) the potential confounders adjusted for were different in each study ; (2) the definition of sleep duration is inconsistent ; (3) the age group of participants varied from study to study;(4)the percentage of participants with long sleep duration.

Compared with previous studies, our study conducted a more detailed relationship analysis of nocturnal sleep time and depressive symptoms in different age and gender groups. We found that those with nocturnal sleep duration of fewer than 7 h with aged<45 years old had a 91% increased risk of depression (P<0.01) compared to participants with 7 h ≤ nocturnal sleep < 9 h, and the strength of association was stronger than those age 45–59 (OR = 1.44) and aged ≥ 60 (OR = 1.29) groups. Further analysis showed that this relationship was most significant in females aged<45(OR = 2.03).

In the present study, insomnia symptoms were comparable to those used in the Diagnostic and Statistical Manual of Mental Disorders (DSM-V) [21]. Among individuals without apparent physical and mental disorders, the proportion reporting at least one insomnia symptom in the present study (45.5%) was generally higher than that reported in the US population [36] as well as the estimates in some other regions of China [27]. Although previous studies indicated that about one-third of adults (30-36%) report at least one nocturnal insomnia symptom [37], validating the prevalence of insomnia symptoms in the Sichuan Basin is at a high level. Furthermore, our results are in agreement with the previous findings [33, 38] that women had more insomnia symptoms than men.

The results of previous studies on the association between insomnia symptoms and depression symptoms are consistent with our study. A few prospective studies, including the US-based HUNT study of 24,715 people, reported that insomnia is a predictive factor of long-term risk of mental diseases [7]. A meta-analysis including 34 prospective studies identified a positive relationship between insomnia symptoms and depression symptoms, the pooled RR was 2.27 [15]. A cross-sectional study of older women over 60 in Shanghai showed that poor sleep quality increases the risk of normal depression symptoms [14]. A co-twin control study showed that depressive symptoms were associated with several objectively measured indices of sleep disturbance [16].

In our study, we found that those in the 45–59 age group with insomnia symptoms had the highest risk of depression symptoms(OR = 2.23), and the risk of women(OR = 2.17) was higher than men(OR = 1.33). Further analysis showed that this relationship was most significant in males aged 45–59(OR = 2.53).

There are several possible explanations for the link between sleep states and depressive symptoms. First, short sleep duration can lead to daytime physical exhaustion, resulting in daytime sleepiness and lethargy, altered circadian rhythms, and increased risk of depression symptoms [39]. Second, inflammation is a key factor strongly associated with depression. The study reported that short sleep duration and insomnia symptoms were associated with increases in inflammatory cytokines such as CRP and IL6 [40, 41]. Third, good sleep helps boost levels of melatonin [42], a molecule that regulates pleiotropic effects that reduce symptoms of depression [43]. Our findings showed the association in females was stronger compared with males, which was consistent with the previous study [28]. In our study, both the prevalence of depression and the association between sleep duration, insomnia symptoms and depression symptoms were higher in females than in males. This may be due to the contribution of sleep duration in women to the onset of depression in the synergistic effect of sex hormones (e.g., estrogen). In addition, females are more likely to experience depression in the context of hormonal fluctuations, during adolescence, pre menstruation, postpartum, and the transition to perimenopause [38]. Notably, in contrast to the previous literature, which focused more on older adults, the 30–45 age group and 45–59 age group in our study showed a stronger association between sleep and depression. It may be due to the reason of young and middle-aged people have greater life and financial pressures. Therefore, more evidence studies on the mechanism of different strength relationships between sleep and depressive symptoms in young and middle-aged people are needed.

The strengths of this study were the large sample size and included many potential confounding factors, such as demographic, socioeconomic information, lifestyles, self-rated health status, pain rate, family social capital, community/society social capital, stressful life events, history of hypertension, and history of diabetes. In addition, our study was more comprehensive because we performed a stratified analysis to examine differences in the relationship between sleep and depressive symptoms by age and gender. However, this study has some limitations. First, the study was cross-sectional, so it was impossible to infer causality. Second, the study population was voluntary, and individuals with major depressive symptoms were less likely to complete the study, which may be another reason for the low prevalence. Third, we used the PHQ-2 to measure depressive symptoms. The scale was suitable for use in community epidemiological studies, and its reliability and validity were demonstrated in this population-friendly study, without clinical knowledge. However, symptoms of depression can be overestimated or underestimated. Participants with positive screening results should be assessed using clinical diagnostic tools or interviewed directly with a clinical psychologist to determine if they met the criteria for major depressive symptoms.

## Conclusion

In summary, nocturnal sleep duration and insomnia symptoms were positively associated with the risk of depressive symptoms in Chinese adults of Sichuan Basin. In three age groups, short-sleep individuals had an increased risk of depression symptoms compared to those with moderate sleep duration, and the same trend was also found in insomniacs compared to non-insomniac, with the strongest associations in the < 45 years age group and 45–59 age group, respectively. In addition, short sleep duration and insomnia were risk factors for depressive symptoms for males and females, and the strength of association in females was stronger compared with males. Moreover, the association between short sleep duration and depression symptoms was highest among women aged less than 45 years, and the association between insomnia symptoms and depression symptoms was the highest among men aged 45–59 years. Our result indicated that short sleep duration and insomnia symptoms are likely signals of depressive symptoms, especially in the middle-aged population and females, which should be more concerned.

## Data Availability

Our study relied on data from China Multi-Ethnic Cohort Study. The summary dataset used during the current study is available from the corresponding author on a reasonable request.

## References

[1] Prevalence, GDaIIa. Collaborators: global, regional, and national incidence, prevalence, and years lived with disability for 354 diseases and injuries for 195 countries and territories, 1990–2017: a systematic analysis for the global burden of Disease Study 2017. Lancet (London England). 2018;392(10159):1789–858. 10.1016/s0140-6736(18)32279-7. 10.1016/S0140-6736(18)32279-7 PMC6227754 30496104

[2] Liu TZ, Xu C, Rota M, Cai H, Zhang C, Shi MJ, Yuan RX, Weng H, Meng XY, Kwong JS (2017). Sleep duration and risk of all-cause mortality: a flexible, non-linear, meta-regression of 40 prospective cohort studies. Sleep Med Rev.

[3] Covassin N, Singh P (2016). Sleep Duration and Cardiovascular Disease Risk: epidemiologic and experimental evidence. Sleep Med Clin.

[4] Smiley A, King D, Bidulescu A. The Association between Sleep Duration and metabolic syndrome: the NHANES 2013/2014. Nutrients. 2019;11(11). 10.3390/nu11112582. 10.3390/nu11112582 PMC6893635 31717770

[5] Léger D, Poursain B, Neubauer D, Uchiyama M (2008). An international survey of sleeping problems in the general population. Curr Med Res Opin.

[6] Sateia MJ (2014). International classification of sleep disorders-third edition: highlights and modifications. Chest.

[7] Sivertsen B, Lallukka T, Salo P, Pallesen S, Hysing M, Krokstad S, Simon Ø (2014). Insomnia as a risk factor for ill health: results from the large population-based prospective HUNT study in Norway. J Sleep Res.

[8] Sofi F, Cesari F, Casini A, Macchi C, Abbate R, Gensini GF (2014). Insomnia and risk of cardiovascular disease: a meta-analysis. Eur J Prev Cardiol.

[9] Sun X, Zheng B, Lv J, Guo Y, Bian Z, Yang L, Chen Y, Fu Z, Guo H, Liang P (2018). Sleep behavior and depression: findings from the China Kadoorie Biobank of 0.5 million chinese adults. J Affect Disord.

[10] Mohan J, Xiaofan G, Yingxian S (2017). Association between sleep time and depression: a cross-sectional study from countries in rural northeastern China. J Int Med Res.

[11] Zhang XF, Liu F, Liu WP, Ye XM, Cui BY, Wang HJ (2021). Relationship between sleep duration and depressive symptoms in middle-aged and elderly people in four provinces of China. Zhonghua Liu Xing Bing Xue Za Zhi.

[12] Li Y, Wu Y, Zhai L, Wang T, Sun Y, Zhang D (2017). Longitudinal Association of Sleep Duration with depressive symptoms among middle-aged and older chinese. Sci Rep.

[13] Berger AT, Wahlstrom KL, Widome R (2019). Relationships between sleep duration and adolescent depression: a conceptual replication. Sleep Health.

[14] Ding L, Zhang L, Cui Y, Gong Q, Ma J, Wang Y, Sang H (2022). The association of sleep duration and quality with depressive symptoms in older chinese women. PLoS ONE.

[15] Li L, Wu C, Gan Y, Qu X, Lu Z (2016). Insomnia and the risk of depression: a meta-analysis of prospective cohort studies. BMC Psychiatry.

[16] Huang M, Bliwise DL, Hall MH, Johnson DA, Sloan RP, Shah A, Goldberg J, Ko YA, Murrah N, Levantsevych OM (2022). Association of depressive symptoms with sleep disturbance: a co-twin control study. Ann Behav Med.

[17] Zeng LN, Zong QQ, Yang Y, Zhang L, Xiang YF, Ng CH, Chen LG, Xiang YT (2020). Gender difference in the prevalence of Insomnia: a Meta-analysis of Observational Studies. Front Psychiatry.

[18] Xiaofan Z, Feng L, Wanpu L, Xianming Y, Binyin C, Huijun W. Relationship between sleep duration and depressive symptoms in middle-aged and elderly people in four provinces of China. *CHINESE JOURNAL OF EPIDEMIOLOGY* 2021, v.42;No.11(11):1955–1961. 10.3760/cma.j.cn112338-20200930-01210. 10.3760/cma.j.cn112338-20200930-01210 34818840

[19] Zhao X, Hong F, Yin J, Tang W, Zhang G, Liang X, Li J, Cui C, Li X (2021). Cohort Profile: the China multi-ethnic cohort (CMEC) study. Int J Epidemiol.

[20] Hirshkowitz M, Whiton K, Albert SM, Alessi C, Bruni O, DonCarlos L, Hazen N, Herman J, Adams Hillard PJ, Katz ES (2015). National Sleep Foundation’s updated sleep duration recommendations: final report. Sleep Health.

[21] Battle DE (2013). Diagnostic and statistical Manual of Mental Disorders (DSM). Codas.

[22] Kroenke K, Spitzer RL, Williams JB (2003). The Patient Health Questionnaire-2: validity of a two-item depression screener. Med Care.

[23] Löwe B, Kroenke K, Gräfe K (2005). Detecting and monitoring depression with a two-item questionnaire (PHQ-2). J Psychosom Res.

[24] Luo Z, Li Y, Hou Y, Liu X, Jiang J, Wang Y, Liu X, Qiao D, Dong X, Li R (2019). Gender-specific prevalence and associated factors of major depressive disorder and generalized anxiety disorder in a chinese rural population: the Henan rural cohort study. BMC Public Health.

[25] Yang S, Gao B, Gu J, Gong Y, Yu B, Han J, Dong P, Jia P, Yang S (2020). Relationship between social capital and heroin use behaviors among patients in methadone maintenance treatment in Sichuan Province, China: a cross-sectional study. Med (Baltim).

[26] Han J, Jia P, Huang Y, Gao B, Yu B, Yang S, Yu J, Xiong J, Liu C, Xie T (2020). Association between social capital and mental health among older people living with HIV: the Sichuan Older HIV-Infected cohort study (SOHICS). BMC Public Health.

[27] Chen Y, Kartsonaki C, Clarke R, Guo Y, Yu C, Bian Z, Jiang Q, Li S, Chen J, Li L (2018). Characteristics and correlates of sleep duration, daytime napping, snoring and insomnia symptoms among 0.5 million chinese men and women. Sleep Med.

[28] Ouyang P, Sun W (2019). Depression and sleep duration: findings from middle-aged and elderly people in China. Public Health.

[29] Sun Y, Shi L, Bao Y, Sun Y, Shi J, Lu L (2018). The bidirectional relationship between sleep duration and depression in community-dwelling middle-aged and elderly individuals: evidence from a longitudinal study. Sleep Med.

[30] Zhai L, Zhang H, Zhang D, SLEEP DURATION AND, DEPRESSION AMONG ADULTS (2015). A META-ANALYSIS OF PROSPECTIVE STUDIES. Depress Anxiety.

[31] Paudel M, Taylor BC, Ancoli-Israel S, Blackwell T, Maglione JE, Stone K, Redline S, Ensrud KE (2013). Sleep disturbances and risk of Depression in older men. Sleep.

[32] Yokoyama E, Kaneita Y, Saito Y, Uchiyama M, Matsuzaki Y, Tamaki T, Munezawa T, Ohida T (2010). Association between depression and insomnia subtypes: a longitudinal study on the elderly in Japan. Sleep.

[33] Gehrman P, Seelig AD, Jacobson IG, Boyko EJ, Hooper TI, Gackstetter GD, Ulmer CS, Smith TC (2013). Predeployment sleep duration and insomnia symptoms as risk factors for New-Onset Mental Health Disorders following Military Deployment. Sleep.

[34] Dong L, Xie Y, Zou X (2022). Association between sleep duration and depression in US adults: a cross-sectional study. J Affect Disord.

[35] Supartini A, Oishi T, Yagi N. Sex differences in the relationship between Sleep Behavior, Fish Consumption, and depressive symptoms in the General Population of South Korea. Int J Environ Res Public Health. 2017;14(7). 10.3390/ijerph14070789. 10.3390/ijerph14070789 PMC5551227 28708121

[36] Ohayon MM (2002). Epidemiology of insomnia: what we know and what we still need to learn. Sleep Med Rev.

[37] Morin CM, Jarrin DC (2022). Epidemiology of Insomnia: prevalence, course, risk factors, and Public Health Burden. Sleep Med Clin.

[38] La YK, Choi YH, Chu MK, Nam JM, Choi YC, Kim WJ (2020). Gender differences influence over insomnia in korean population: a cross-sectional study. PLoS ONE.

[39] Luca A, Luca M, Calandra C (2013). Sleep disorders and depression: brief review of the literature, case report, and nonpharmacologic interventions for depression. Clin Interv Aging.

[40] Lee YC, Son DH, Kwon YJ. U-Shaped Association between Sleep Duration, C-Reactive protein, and Uric Acid in Korean Women. Int J Environ Res Public Health. 2020;17(8). 10.3390/ijerph17082657. 10.3390/ijerph17082657 PMC7216061 32294936

[41] Kim S, Yoon H (2020). Volunteering, subjective sleep quality, and chronic inflammation: a 5-Year Follow-Up of the National Social Life, Health, and Aging Project. Res Aging.

[42] Yaşar NF, Badak B, Canik A, Baş S, Uslu S, Öner S, Ateş E. Effects of Sleep Quality on Melatonin Levels and Inflammatory Response after Major Abdominal Surgery in an Intensive Care Unit. Molecules2017, 22(9) 10.3390/molecules22091537 PMC6151787 28895895

[43] Satyanarayanan SK, Su H, Lin YW, Su KP (2018). Circadian rhythm and melatonin in the treatment of Depression. Curr Pharm Des.

